# Unsupervised Optical-Sensor Extrinsic Calibration via Dual-Transformer Alignment

**DOI:** 10.3390/s25226944

**Published:** 2025-11-13

**Authors:** Yuhao Wang, Yong Zuo, Yi Tang, Xiaobin Hong, Jian Wu, Ziyu Bian

**Affiliations:** 1 School of Electronic Engineering, Beijing University of Posts and Telecommunications, Beijing 100876, China; 1648541693@bupt.edu.cn (Y.W.); xbhong@bupt.edu.cn (X.H.); jianwu@bupt.edu.cn (J.W.); 2 School of Optics and Photonics, Beijing Institute of Technology, Beijing 100876, China; 3220235092@bit.edu.cn

**Keywords:** LiDAR–camera calibration, unsupervised, sensor fusion, extrinsic parameters

## Abstract

Accurate extrinsic calibration between optical sensors, such as camera and LiDAR, is crucial for multimodal perception. Traditional methods based on specific calibration targets exhibit poor robustness in complex optical environments such as glare, reflections, or low light, and they rely on cumbersome manual operations. To address this, we propose a fully unsupervised, end-to-end calibration framework. Our approach adopts a dual-Transformer architecture: a Vision Transformer extracts semantic features from the image stream, while a Point Transformer captures the geometric structure of the 3D LiDAR point cloud. These cross-modal representations are aligned and fused through a neural network, and a regression algorithm is used to obtain the 6-DoF extrinsic transformation matrix. A multi-constraint loss function is designed to enhance structural consistency between modalities, thereby improving calibration stability and accuracy. On the KITTI benchmark, our method achieves a mean rotation error of 0.21° and a translation error of 3.31 cm; on a self-collected dataset, it attains an average reprojection error of 1.52 pixels. These results demonstrate a generalizable and robust solution for optical-sensor extrinsic calibration, enabling precise and self-sufficient perception in real-world applications.

## 1. Introduction

Multi-modal perception has become a cornerstone of modern autonomous systems, where complementary information from optical sensors such as LiDAR and cameras is integrated to achieve reliable scene understanding. A critical step in this integration is extrinsic calibration, which aligns geometric structures captured by LiDAR with semantic-rich visual cues from cameras. Accurate calibration provides the geometric foundation for transforming and fusing heterogeneous sensor data, enabling downstream tasks such as 3D object detection, semantic mapping, and autonomous navigation.

Conventional calibration approaches predominantly rely on supervised methods involving calibration targets (e.g., checkerboards or AprilTags) [1,2], manual annotation, or precise sensor synchronization. To improve calibration accuracy, Geiger [3] introduced an automated calibration system utilizing multiple checkerboards as targets. This strategy introduces redundancy into the calibration process, thereby enhancing both stability and precision. To further reduce edge detection errors in sparse point clouds, Tóth [4] proposed the use of spherical calibration targets. Their continuous and symmetric surfaces enable reliable reconstruction in both LiDAR and camera modalities. These target-based methods are effective under limited conditions; however, they still require cumbersome manual operations, are susceptible to environmental factors, and are unsuitable for real-time or large-scale deployment in dynamic environments.

To reduce manual effort and improve scalability, recent research has explored unsupervised and weakly supervised calibration frameworks. These methods often leverage photometric consistency, mutual information, or self-supervised feature correspondences to align point clouds and images without ground-truth annotations. For instance, Yuan et al. [5] proposed an efficient edge extraction technique based on voxel segmentation and plane fitting, while Liu et al. [6] employed geometric features such as line segments and rectangles extracted from urban scenes to maximize 2D–3D feature correspondences. Although these approaches reduce reliance on manual labeling, they still struggle with generalization, robustness, and accuracy in complex or unstructured environments.

To overcome these challenges, deep learning (DL)-based calibration methods have recently gained traction. Early approaches such as RegNet [7] applied convolutional neural networks (CNNs) to regress extrinsic parameters using Euclidean loss, marking a shift from heuristic to data-driven calibration. Zhu et al. [8] further redefined calibration as an optimization task and proposed semantic-driven metrics to evaluate alignment quality. Unlike traditional pipelines, DL-based methods achieve higher accuracy with minimal manual intervention. However, most existing models rely on RGB–depth fusion or shallow geometric cues, which capture incomplete and poorly aligned information, and they rarely exploit global feature extraction across modalities. This limits both calibration accuracy and generalization capability.

In this work, we propose a fully unsupervised and end-to-end framework for LiDAR–camera extrinsic calibration that eliminates the need for manual intervention, handcrafted features, or prior synchronization. Our method builds on a Transformer-based framework [9] to extract and align global features from 2D images and 3D point clouds: a Vision Transformer encodes rich semantic features from RGB images, while a Point Transformer captures spatial structure from LiDAR data. The extracted features are fused and processed through a regression network to estimate the 4 × 4 extrinsic transformation matrix. Moreover, we design a multi-constraint loss function that enforces structural consistency while improving the stability and physical validity of extrinsic calibration. Extensive experiments on both the KITTI dataset [10] and our self-collected dataset validate the effectiveness of the proposed method. In summary, the main contributions of this work lie in:

(1) A dual-Transformer framework for unsupervised cross-modal feature extraction and alignment;
(2) A multi-constraint loss function that enhances geometric consistency and calibration stability;
(3) Comprehensive validation on both benchmark and real-world data, demonstrating the scalability and generalizability of the proposed approach.

## 2. Methods

In this section, we present a fully unsupervised LiDAR–camera extrinsic calibration framework based on a Transformer-based architecture. The framework is designed to automatically infer the spatial transformation between LiDAR and camera sensors without requiring manual annotations. The overall pipeline consists of three main components: (1) an image encoder based on the Vision Transformer [11], which extracts global semantic features from 2D images; (2) a Point Transformer module, which captures geometric representations from 3D point clouds and models the spatial structure of the environment; and (3) a transformation regression module, which fuses the extracted features and estimates the 4 × 4 extrinsic transformation matrix between the two sensors.

**Figure 1:**
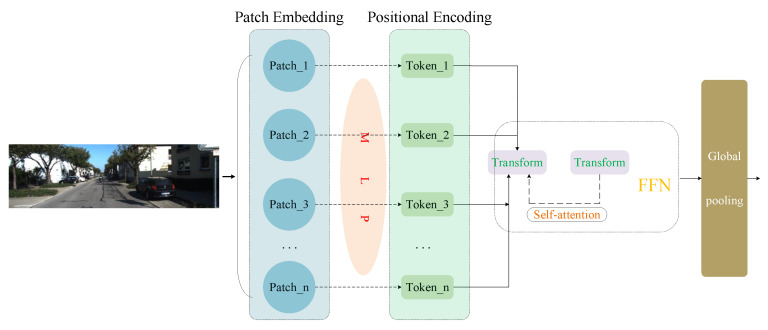
Two-dimensional feature extraction architecture diagram.

For image feature extraction, we employ a Vision Transformer encoder [11], as illustrated in Figure 1. The input RGB image is resized to 512 × 512 to ensure a compact yet sufficiently detailed representation, and then divided into non-overlapping 16 × 16 patches, yielding 1024 tokens. Each patch is flattened and projected by a multilayer perceptron (MLP) into a 1024-dimensional embedding. To preserve spatial information, learnable positional encodings are added, and the resulting token sequence is processed by 8 Transformer layers, each consisting of multi-head self-attention (with 8 heads) and feed-forward networks (hidden dimension 2048). Finally, a global pooling operation aggregates the outputs into a compact feature descriptor that provides the 2D semantic representation for cross-modal alignment with 3D point cloud features.

The choice of these parameters follows a balance between representation capacity and computational efficiency. The 512 × 512 input resolution provides sufficient detail while keeping computation tractable; a patch size of 16 maintains a reasonable granularity of spatial information. The embedding dimension of 1024 and 8 attention heads allow the model to capture rich dependencies across image regions without excessive overhead, while the feed-forward dimension of 2048 follows standard Transformer design for stable training and expressive features. The use of 8 Transformer layers further provides adequate depth for semantic modeling while avoiding overfitting under unsupervised training.

The Transformer-based encoder provides several advantages for calibration tasks. First, its ability to capture global dependencies enhances robustness in complex scenes and improves the accuracy of cross-modal alignment. Second, its flexible design naturally facilitates integration with 3D point cloud features, enabling more reliable calibration across modalities.

In the calibration process, the extraction of 3D features plays a crucial role in determining the accuracy, stability, and adaptability of the results. Compared with traditional methods that rely on sparse point or line features, global 3D features provide richer structural information, enabling more robust feature matching in structured environments and multi-sensor fusion scenarios. Robust 3D feature extraction also mitigates the influence of environmental changes, occlusions, and noise, thereby improving the stability of cross-sensor alignment.

**Figure 2:**
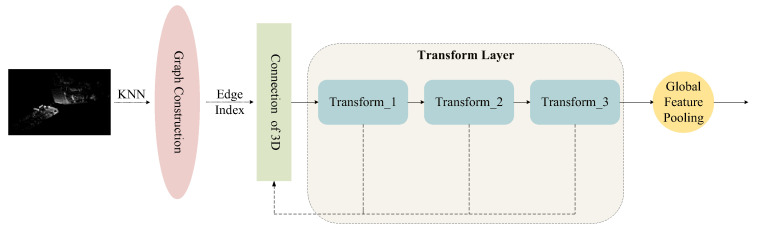
Three-dimensional feature extraction architecture diagram.

Based on these considerations, we adopt a Transformer-based approach for 3D feature extraction. The self-attention mechanism enables modeling of both local neighborhoods and long-range dependencies, allowing the network to capture complete geometric structures and establish robust feature correspondences across modalities. The 3D feature extraction process in our framework, illustrated in Figure 2, consists of the following four steps:

**A. KNN Graph Construction:** Since point clouds are inherently unordered sets of discrete points, they lack explicit structural information for direct feature extraction. To address this, we employ the k-nearest neighbors (kNN) [12] algorithm to construct a local topological structure. Specifically, for each point, its nearest neighbors are identified by computing Euclidean distances in the 3D coordinate space, where efficient search methods such as k-d tree indexing are used to accelerate neighbor retrieval. A sparse graph is then formed, in which points serve as nodes and neighbor relations define edges, enabling the model to capture local geometric structures. We set k=8, based on a balance between preserving sufficient local geometric information and avoiding the inclusion of noisy or redundant neighbors. Smaller values of k may lead to incomplete neighborhood representation, while larger values increase computational cost and risk incorporating irrelevant points.

**B. Adjacency Matrix Generation and Computational Optimization:** Following the KNN computation, an adjacency matrix (edge index) is generated, representing the connectivity between points. This adjacency matrix not only structures the point cloud into a graph but also constrains the computation of the Transformer to local neighborhoods, significantly reducing computational complexity.

The attention weight between point i and one of its neighbors j is dynamically computed as [13,14]:

(1) $$\alpha_{ij}=Soft\max(q_{i}^{T}k_{j})$$

where $q_{i}$ and $k_{j}$ are the query and key feature embeddings of point i and j, respectively. The attention mechanism is built upon this adjacency information, ensuring that each point can effectively aggregate features from its neighboring points, thereby transforming the point cloud from an unstructured set into a structured graph for more efficient feature extraction.

**C. Transformer-based Feature Extraction:** Once the local neighborhood graph is established, the point cloud is fed into a Transformer module for feature extraction. During this stage, each point’s 3D coordinates are first projected into a 256-dimensional embedding, providing a compact yet expressive representation. The token sequence is then processed by 4 Transformer layers, each consisting of multi-head self-attention with 4 heads and a feed-forward network of hidden dimension 512. These parameters were chosen to strike a balance between representation capacity and computational efficiency: the 256-dimensional embedding offers sufficient expressiveness for local and global geometric modeling, while the use of 4 layers and 4 heads provides adequate modeling depth and multi-subspace feature learning without incurring excessive computational cost.

Leveraging the self-attention mechanism, the Transformer models the relationships between points within local neighborhoods while also capturing long-range dependencies across distant points. Unlike conventional methods or CNN-based approaches that rely on fixed local receptive fields, the Transformer enables adaptive learning of multi-level feature representations across different subspaces. This allows even spatially distant points to establish effective global connections, thereby enhancing the completeness and robustness of point cloud representations. Additionally, the Transformer incorporates an adaptive weighting mechanism that dynamically adjusts the contribution of neighboring points during feature aggregation [15]. Specifically, the aggregated feature of point i is computed as:

(2) $$f_{i}^{agg}=\sum_{j\in N(i)}\alpha_{ij}v_{j}$$

where $v_{j}$ denotes the value embedding of neighbor j, and $\alpha_{ij}$ is the learned attention weight derived from the Softmax operation. This formulation allows informative and geometrically relevant neighbors to contribute more strongly, while suppressing noisy or less relevant points, enabling the model to better handle irregular spatial distributions in real-world point clouds.

**D. Global Feature Aggregation:** To obtain a comprehensive global representation of the entire point cloud, we apply Global Average Pooling (GAP) [16] over all points’ output features from the Transformer. This process aggregates individual point features into a single global feature vector that encapsulates the overall spatial structure and semantic information of the point cloud. The resulting global feature serves as a compact and expressive representation, supporting subsequent multi-modal fusion and transformation regression tasks.

**Figure 3:**
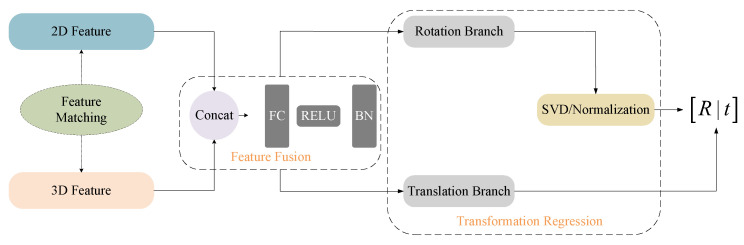
Regression Flowchart.

As illustrated in Figure 3, our method first extracts global features from both the image and the point cloud. These features encode semantic information from the image and geometric structure from the point cloud. To enable cross-modal interaction, both features are projected into a shared 512-dimensional embedding space using two learnable linear layers. After projection, the 2D and 3D embeddings are concatenated and passed through MLP function [17], producing a unified cross-modal feature representation. Compared to raw-level alignment, this high-level semantic fusion is more robust to noise, occlusion, and viewpoint changes.

After feature alignment, the features from both modalities are concatenated into a 1024-dimensional vector and passed into the feature fusion module, which is implemented as a lightweight multi-layer perceptron composed of fully connected layers, ReLU activation, and batch normalization. This design allows the network to perform deep-level fusion of the concatenated features, ensuring that the complementary semantics from the image and the geometric cues from the point cloud are effectively integrated into a unified cross-modal representation.

The fused features are fed into a transformation regression module consisting of two parallel branches [18]: a Rotation Branch and a Translation Branch. 

In the Rotation Branch, the network first outputs an intermediate matrix $\hat{R}\in {\mathbb{R}}^{3\times 3}$. To guarantee geometric validity, $\hat{R}$ is converted into a proper orthogonal rotation matrix through Singular Value Decomposition (SVD). Specifically,

(3) $$\hat{R}=U\Sigma V^{T},\;R=UV^{T}$$

where U and V are orthogonal matrices, and Σ is a diagonal singular-value matrix, which enforces $R^{T}R=I,\;\;\;\det(R)=1$. This SVD-based normalization prevents drift and ensures a stable rotation representation during training.

In the Translation Branch, the network directly regresses a 3D translation vector $t={\left[t_{x},t_{y},t_{z}\right]}^{T}$, representing the displacement between the two sensor coordinate systems.

Finally, the rotation R and translation t are combined to form the 6-DoF rigid transformation from the camera frame to the LiDAR frame:

(4) $$T=\left[\begin{array}{cc} R & t\\ 0 & 1 \end{array}\right]$$

After obtaining the extrinsic parameters, the transformation matrix is applied to project the 3D point cloud onto the image plane, thereby establishing spatial correspondence between the 3D geometric features and the 2D appearance features. This step not only provides a clear physical interpretation of cross-modal alignment but also offers natural guidance for subsequent optimization, enabling the framework to progressively improve accuracy and stability under unsupervised conditions without the need for manual annotations or additional calibration targets.

This mechanism seamlessly integrates feature extraction, semantic-level alignment, feature fusion, and transformation regression. Feature extraction ensures discriminative representations, feature fusion achieves effective modality integration, the regression network captures the cross-modal spatial mapping, and the projection operation provides inherent supervisory signals. This end-to-end design enhances the automation of the calibration process and demonstrates strong robustness and adaptability in complex environments, offering an efficient and annotation-free solution for camera–LiDAR calibration.

## 3. Results

In this section, we conduct experiments to validate the proposed unsupervised LiDAR–camera calibration framework. The evaluation covers multiple datasets and experimental settings, including loss design, quantitative and qualitative results, and ablation studies to analyze the contribution of each component.

### 3.1. Dataset

We evaluate the proposed method on both the KITTI dataset and a self-collected dataset.

KITTI dataset: The KITTI benchmark provides RGB–LiDAR pairs acquired in outdoor driving scenarios. Each sample consists of a high-resolution left camera image and a corresponding Velodyne point cloud. The ground-truth extrinsic parameters are only used for evaluation and visualization, ensuring that our framework remains fully unsupervised during training. Images are resized and normalized before being fed into the Vision Transformer, while point clouds are filtered to remove distant or low-intensity points. From the remaining points, 4096 are randomly sampled to maintain computational efficiency and uniformity.

Self-collected dataset: To further validate generalization in real-world applications, we constructed a dataset using a Livox Horizon LiDAR (Livox Technology Company Limited, Hong Kong, China) and an MV-CS050-10GC camera (HIKROBOT, Hangzhou, China) in outdoor environments. This dataset covers more diverse sensor configurations and environmental conditions compared with KITTI. Similar preprocessing is applied, including image normalization and random sampling of 4096 LiDAR points.

### 3.2. Loss Function

In our framework, we design a multi-constraint loss function to enforce structural consistency and ensure the physical validity of the predicted extrinsic parameters. The overall loss is defined as:

(5) $$L_{\mathrm{total}}=\lambda_{struct}\cdot L_{struct}+\lambda_{ortho}\cdot L_{ortho}+\lambda_{trans}\cdot L_{trans}$$

where $\lambda_{struct}$, $\lambda_{ortho}$ and $\lambda_{trans}$ are weighting factors that balance the contributions of different loss terms. $L_{struct}$ denotes the structural consistency loss; $L_{ortho}$ is the rotation orthogonality loss; $L_{trans}$ is the translation regularization loss.

- Structural Consistency Loss ($L_{struct}$)

To enforce geometric alignment between the image and the point cloud in an unsupervised manner, we define a structural consistency loss [19]. Specifically, a 3D LiDAR point $p_{i}$ is first transformed into the camera coordinate system using the estimated extrinsics:

(6) $$p_{i}^{cam}=R\cdot p_{i}+t={\left[x_{i}^{cam},y_{i}^{cam},z_{i}^{cam}\right]}^{T}$$

where (R,t) denote the predicted rotation and translation matrices, and $p_{i}^{cam}$ denotes the 3D LiDAR point represented in the camera coordinate system. To project this 3D point to the image plane, perspective projection is applied:

(7) $$u_{i}^{norm}=\frac{u^{cam}}{w^{cam}},v_{i}^{norm}=\frac{v^{cam}}{w^{cam}}$$

(8) $$p_{i}^{proj}=K\left[\begin{array}{l} u_{i}^{norm}\\ v_{i}^{norm}\\ 1 \end{array}\right]=\left[\begin{array}{l} u_{i}\\ v_{i}\\ 1 \end{array}\right]$$

$p_{i}^{proj}$ denotes the 2D projection of the i-th 3D point onto the image plane under the predicted transformation. Thus, $\left(u_{i},v_{i}\right)$ are the 2D pixel coordinates corresponding to point $p_{i}$.

To enforce cross-modal geometric alignment, we compare the semantic feature of $p_{i}$ in the point cloud with the feature sampled at $\left(u_{i},v_{i}\right)$ on the image feature map:

$f_{pc}(p_{i})$: 3D point feature extracted by the point cloud Transformer.

$f_{img}(u_{i},v_{i})$: 2D semantic feature obtained by bilinear interpolation on the image feature map.

Finally, the structural consistency loss is defined as:

(9) $$L_{\mathrm{struct}}=\frac{1}{N}\sum_{i=1}^{N}\left\|f_{\mathrm{img}}\left(p_{i}^{proj}\right)-f_{\mathrm{pc}}(p_{i})\right\|$$

$f_{\mathrm{img}}\left(p_{i}^{proj}\right)$ represents the image feature sampled at the projected location on the image feature map; $f_{\mathrm{pc}}(p_{i})$ refers to the point cloud feature corresponding to the original 3D point.

This loss forces projected LiDAR points to be consistent with their visual counterparts, enabling geometry-aware supervision without ground-truth extrinsics.

- Rotation Orthogonality Loss ($L_{ortho}$)

To ensure that the predicted rotation matrix R represents a physically valid 3D rotation, we enforce its orthogonality [20]:

(10) $$L_{ortho}=||R^{T}R-I|{|}_{F}$$

This ensures physically meaningful and interpretable rotations.

- Translation Regularization Loss ($L_{trans}$)

To discourage unrealistic translations, we penalize the $L_{2}-norm$ of the predicted translation vector t:

(11) $$L_{trans}=||t|{|}_{2}^{2}$$

This improves stability and constrains predictions to realistic sensor configurations. The translation regularization loss is assigned only a weak weight and serves to discourage excessively large values. A trivial solution t=0 is avoided because the reprojection consistency loss dominates the optimization, ensuring physically meaningful, non-zero translations.

To ensure a closed optimization loop, the entire network is trained in an end-to-end manner. The total loss $L_{\mathrm{total}}$ is computed as the weighted sum of all loss terms and is backpropagated through all learnable parameters, ensuring that both the extrinsic transformation and feature extraction modules are jointly optimized.

For implementation details, we use the Adam optimizer with an initial learning rate of $1\times 10^{-4}$. The model is trained with a batch size of 8 for 200 epochs on the KITTI dataset. All modules are trained jointly from random initialization, ensuring stable convergence and optimal performance.

### 3.3. Experiment

In this subsection, we present the experimental results of the proposed calibration framework. The evaluation is carried out on the KITTI benchmark and our self-collected dataset, followed by ablation studies to investigate the contribution of individual modules.

#### 3.3.1. Calibration on KITTI Dataset

In this section, we evaluate the proposed unsupervised calibration method on the KITTI dataset. KITTI is one of the most representative benchmarks in autonomous driving, providing synchronized LiDAR point clouds, RGB images, and high-precision extrinsic parameters. Specifically, we adopt Sequence 00 of the KITTI odometry dataset, where paired images and point clouds are used to estimate the LiDAR–camera extrinsic transformation, enabling quantitative evaluation of our method without any super-vision. The KITTI benchmark provides ground-truth extrinsic parameters obtained via a target-based calibration procedure conducted during dataset collection. Therefore, the reported rotation and translation errors in our experiments are measured with respect to this benchmark reference. To comprehensively validate the effectiveness of the proposed approach, we compare it against several representative methods, including unsupervised approaches CalibDepth [21] and RegNet, as well as supervised approaches LCCNet [22], CalibNet [23], and CALNet [24].

In our framework, the Vision Transformer encoder extracts semantic features from RGB images, while the Point Transformer models geometric structures from LiDAR point clouds. The extracted 2D and 3D features are fused and passed through a regression network to predict the 6-DoF extrinsic transformation matrix. The predicted transformation is then optimized in an end-to-end manner using the proposed multi-constraint loss, which directly supervises the regression output and enforces cross-modal consistency. Through backpropagation, the Vision Transformer encoder parameters are updated jointly with the Point Transformer and regression module, ensuring that the Vision Transformer contributes directly to the calibration process, see Table 1.

Our method achieves the lowest rotation error among unsupervised approaches, with an average of 0.21° (0.18° roll, 0.36° pitch, 0.13° yaw). For translation, it obtains an average error of 3.31 cm (3.8, 2.2, and 3.9 cm along X, Y, and Z, respectively). Compared with CALNet, a strong supervised baseline (0.18°/2.9 cm), our method delivers competitive performance without using any ground-truth extrinsic supervision.

Relative to other unsupervised methods such as RegNet (0.28°/6.00 cm) and CalibDepth (0.37°/1.56 cm), our model achieves a better trade-off between rotation and translation accuracy. While LCCNet, a supervised approach, yields the best overall accuracy, our results demonstrate that fully unsupervised calibration can still attain competitive performance without prior extrinsic knowledge.

**Figure 4:**
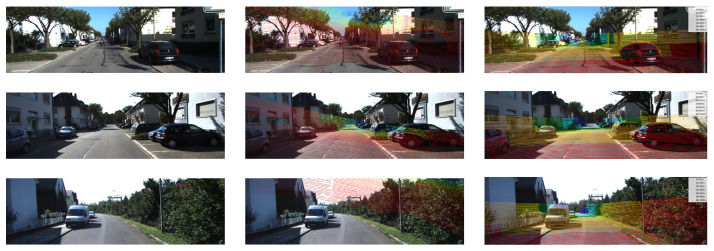
Qualitative results of LiDAR–camera calibration on the KITTI dataset (Original/Uncalibrated/Calibrated with Estimated Extrinsics).

To further validate the effectiveness of our proposed approach, we provide qualitative visualization results of LiDAR-to-image projections under different calibration settings, as shown in Figure 4.

The first column illustrates the original RGB images from the KITTI dataset, serving as the reference for alignment. In the second column, we present the uncalibrated case where the extrinsic parameters are perturbed. In this setting, the projected LiDAR points fail to align with the true scene geometry: points that should fall on solid surfaces such as road boundaries, vehicles, or building facades appear scattered in free space, producing floating points and distorted structures. These severe inconsistencies highlight the necessity of accurate calibration in multi-sensor fusion tasks. In contrast, the third column demonstrates the results obtained using the extrinsic parameters estimated by our framework. In the experimental results in the third column, we color-coded the LiDAR points according to depth ranges: 0–10 m (red), 10–20 m (orange), 20–30 m (yellow), 30–40 m (green), 40–60 m (cyan), 60–80 m (blue), and 80–100 m (purple). Here, the projected LiDAR points exhibit a high degree of spatial consistency with the visual scene. Specifically, road surfaces are clearly delineated, vehicles are tightly outlined, and building contours are precisely matched, indicating that the predicted transformation successfully bridges the geometric gap between the two modalities. Such improvements not only reflect the accuracy of our calibration method but also demonstrate its robustness under real-world scenarios.

These qualitative results confirm that our unsupervised calibration framework can effectively recover precise camera–LiDAR alignment without requiring manual annotation or dedicated calibration targets. By leveraging feature-level correspondence and projection-based consistency, the method ensures reliable multimodal alignment, which is critical for downstream perception tasks such as 3D object detection, semantic segmentation, and scene understanding.

#### 3.3.2. Calibration on Self-Collected Dataset

Beyond experiments on public datasets such as KITTI, we further conducted evaluations on a self-collected dataset to assess the generalization capability of our proposed calibration framework.

Compared with standardized benchmarks, the self-collected data introduces additional challenges such as non-uniform LiDAR scanning patterns, varying illumination conditions, and moving objects. These factors provide a more realistic and rigorous test of the robustness of our calibration method. By analyzing this dataset, we aim to demonstrate that our framework not only achieves accurate calibration on well-structured benchmarks but also maintains stable and reliable performance under unconstrained, real-world conditions.

Since our self-collected dataset does not provide ground-truth extrinsic parameters, absolute rotation and translation errors cannot be computed. Instead, we employ reprojection error as the evaluation metric, measured by projecting LiDAR points onto the image plane and comparing them with detected checkerboard corners from calibration boards. This provides a reliable surrogate for quantitative evaluation in the absence of ground-truth extrinsics.

To further validate the effectiveness of our framework, we conducted a comparative evaluation on the self-collected dataset using several representative calibration methods, including a Canny-based geometric approach, the unsupervised deep learning method CalibDepth, and the supervised deep learning method CALNet. Table 2 summarizes the mean reprojection error of these approaches together with our method. Unlike the KITTI benchmark, our self-collected dataset does not provide ground-truth extrinsic parameters, which makes it infeasible to compute absolute rotation and translation errors. Therefore, we adopt reprojection error as the evaluation metric, since it directly measures the pixel-level alignment between projected LiDAR points and image observations. This metric offers a reliable indicator of calibration quality in the absence of ground-truth extrinsics and reflects the method’s applicability to real-world scenarios where manual calibration labels are unavailable.

As shown in the results, the Canny-based method exhibits the largest error of 6.42 px, indicating its limited robustness in real-world scenarios due to sensitivity to noise and unreliable edge detection. CalibDepth, while unsupervised, improves the performance with an error of 4.89 px, demonstrating better adaptability but still lacking stability under challenging conditions. CALNet, which relies on supervised training with annotated calibration targets, achieves a mean error of 2.84 px, highlighting the advantage of explicit supervision. In comparison, our proposed unsupervised framework achieves the lowest mean reprojection error of 1.52 px, significantly surpassing both unsupervised and supervised baselines. This confirms that our feature alignment and projection-consistency mechanism effectively enhance calibration accuracy, while maintaining the advantages of an unsupervised approach.

In addition to the quantitative results, we further conduct qualitative comparisons to more intuitively validate the effectiveness of the proposed method. Specifically, we present the calibration results from two complementary perspectives: first, by projecting the 3D LiDAR point cloud onto the 2D image plane to examine the consistency between geometric contours and image boundaries; and second, by projecting the 2D image onto the 3D point cloud space to verify the correspondence between color information and structural details. These two forms of visualization provide complementary evidence to directly reflect the accuracy and robustness of the calibration results.

**Figure 5:**
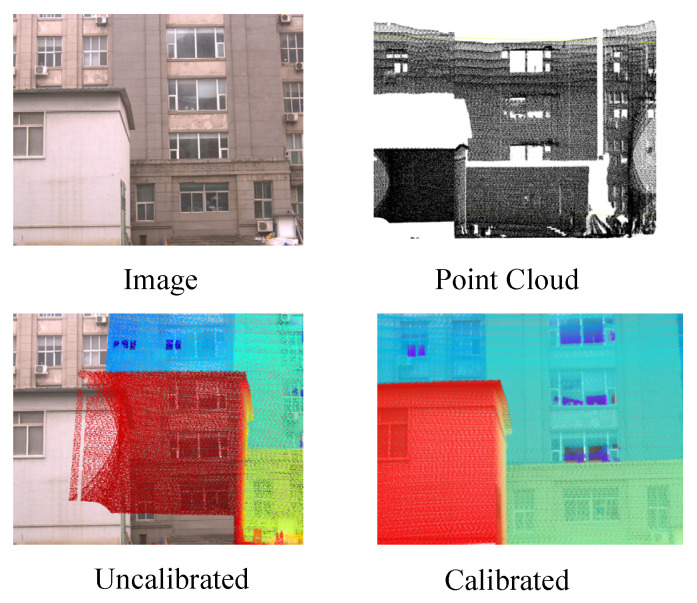
Results of 3D-to-2D projection (Image, Point Cloud, Uncalibrated result, Calibrated result).

- 3D-to-2D projection (LiDAR → Image):

Figure 5 presents the qualitative results of the 3D-to-2D projection, where LiDAR point clouds are projected onto the corresponding RGB image. The figure shows the raw RGB image, the original point cloud, the projection with perturbed extrinsics (Uncalibrated), and the projection using our estimated extrinsics (Calibrated). In the uncalibrated case, significant misalignments can be observed: points from the building façade deviate from the actual window grid, the wall edges appear distorted, and the ground–wall boundary fails to align, resulting in floating and misplaced points. After applying our calibration, the projected points adhere closely to structural boundaries such as window frames, façade contours, and ground surfaces, demonstrating a high degree of spatial consistency between the two modalities. This result highlights the effectiveness of our method in recovering accurate extrinsics and achieving precise geometric alignment between LiDAR and camera data.

**Figure 6:**
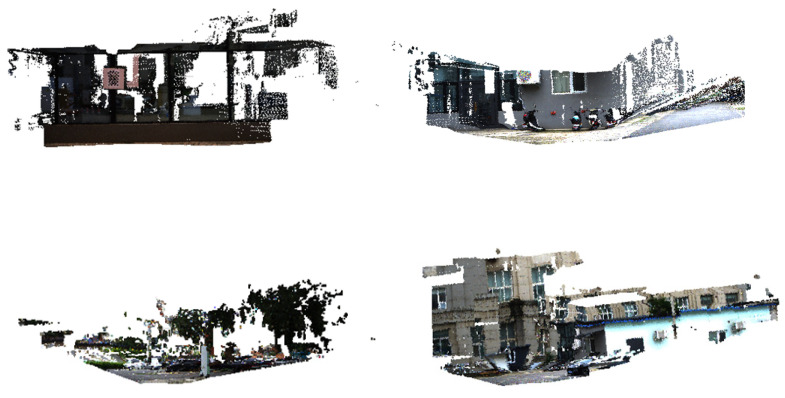
Results of 2D-to-3D projection.

- 2D-to-3D projection (Image → LiDAR):

Figure 6 presents several examples of 2D-to-3D projection results, where image features are reprojected onto the LiDAR point clouds to generate colored 3D reconstructions. Across different scenarios, including building facades, road scenes, and open environments with trees and vegetation, our method consistently demonstrates precise alignment between modalities. For instance, in urban street scenes, structural details such as windows, doors, and walls are accurately mapped onto the corresponding 3D geometry, resulting in point clouds with clear semantic boundaries. In outdoor environments, vegetation and road textures are well preserved, with the projected color distribution tightly adhering to the underlying 3D surfaces. These results highlight the natural fusion of 2D and 3D modalities achieved by our calibration method and further confirm its robustness in delivering strong cross-modal consistency, which is crucial for downstream applications such as semantic mapping, scene reconstruction, and multimodal perception in complex environments.

#### 3.3.3. Ablation Experiment

To further investigate the contribution of each module in our framework, we conduct an ablation study on the KITTI dataset, with results summarized in Table 3. The complete model achieves the best performance with the lowest rotation and translation errors, while removing or altering any component leads to noticeable degradation, confirming the necessity of each design.

When the Point Transformer is removed, the translation error increases from 3.31 cm to 4.82 cm, indicating that fine-grained 3D geometric modeling is crucial for accurate cross-modal alignment. Substituting the Vision Transformer with a standard CNN encoder results in higher rotation error (0.30° vs. 0.21°), which highlights the importance of capturing long-range semantic dependencies from the image domain.

The feature fusion module also proves essential: without it, the integration of image and point cloud features becomes less effective, leading to errors of 5.13 cm in translation and 0.41° in rotation. This shows that deep-level fusion is necessary to exploit complementary cross-modal cues. Finally, removing the multi-constraint loss causes the most significant performance drop (RTE 6.35 cm, RRE 0.53°), demonstrating that appropriate constraints during optimization are indispensable for stable and accurate calibration.

These results confirm that each component—feature extraction, fusion, and loss design—contributes meaningfully to the overall performance, and their combination ensures the robustness and accuracy of our framework.

## 4. Conclusions

In this paper, we proposed a novel unsupervised framework for camera–LiDAR calibration that leverages dual Transformer architectures to extract semantic features from images and geometric structures from point clouds. By introducing a feature matching module, a dedicated feature fusion block, and a regression–projection mechanism, our method effectively learns the spatial mapping between modalities and predicts accurate 6-DoF extrinsic parameters without requiring manual annotations or calibration targets.

Extensive experiments on benchmark datasets and real-world self-collected data demonstrated that our framework achieves state-of-the-art calibration accuracy, with both qualitative visualizations and quantitative evaluations confirming its robustness and adaptability. Ablation studies further verified the necessity of each module, highlighting the complementary roles of feature extraction, feature fusion, and transformation regression in achieving precise cross-modal alignment.

Looking forward, this work opens up several promising directions. One avenue is to extend the framework to online calibration for dynamic environments, enabling real-time adaptation to sensor drift. Another direction is to integrate the calibration process into downstream tasks such as 3D detection, semantic mapping, and scene reconstruction, allowing end-to-end optimization of multimodal perception systems. We believe that the proposed framework provides a solid foundation for advancing autonomous driving and robotics applications by offering a reliable, annotation-free, and scalable solution to camera–LiDAR calibration.

## Figures and Tables

**Table 1:** Quantitative comparison of LiDAR–camera extrinsic calibration on the KITTI dataset (The table reports rotation error (RRE, °) and translation error (RTE, cm), including per-axis results for roll, pitch, yaw, and X, Y, Z.).

Model	Supervision	RRE (°)	Roll (°)	Pitch (°)	Yaw (°)	RTE (cm)	X(cm)	Y(cm)	Z(cm)
CalibDepth	NO	0.37	0.12	0.91	0.14	1.56	1.82	1.28	1.43
RegNet	NO	0.28	0.36	0.25	0.24	6.00	7.0	7.0	4.0
LCCNet	Yes	0.17	0.06	0.05	0.12	2.10	2.3	1.6	2.4
CalibNet	Yes	0.36	0.13	0.78	0.17	6.90	10.4	2.9	7.4
CALNet	Yes	0.18	0.08	0.33	0.13	2.90	3.4	1.4	3.9
Our	NO	0.21	0.18	0.36	0.13	3.31	3.8	2.2	3.9

**Table 2:** Mean reprojection error on the self-collected dataset.

Model	Supervision	Mean Reprojection Error (px)
Canny-based	No	6.42
CalibDepth	No	4.89
CALNet	Yes	2.84
Ours	No	1.52

**Table 3:** Ablation Result on the KITTI dataset.

Model	RTE ↓ (cm)	RRE ↓ (°)
Full (Ours)	3.31	0.21
*w*/*o* Point Transformer	4.82	0.35
*w*/*o* Vision Transformer	4.24	0.30
*w*/*o* Feature Fusion Module	5.13	0.41
*w*/*o* Reprojection Consistency Loss	6.35	0.53

## Data Availability

The datasets used in this study include both publicly available and self-collected data. The public KITTI dataset is accessible at http://www.cvlibs.net/datasets/kitti. The self-collected datasets are not publicly available due to equipment limitations and privacy considerations, but they are available from the corresponding author upon reasonable request.

## References

[1] Kassir A., Peynot T. (2010). Reliable automatic camera-laser calibration. Proceedings of the 2010 Australasian Conference on Robotics & Automation.

[2] Zhou L., Deng Z. (2012). Extrinsic calibration of a camera and a lidar based on decoupling the rotation from the translation. Proceedings of the 2012 IEEE Intelligent Vehicles Symposium.

[3] Geiger A., Moosmann F., Car Ö., Schuster B. (2012). Automatic camera and range sensor calibration using a single shot. Proceedings of the 2012 IEEE International Conference on Robotics and Automation.

[4] Tóth T., Pusztai Z., Hajder L. (2020). Automatic LiDAR-camera calibration of extrinsic parameters using a spherical target. Proceedings of the 2020 IEEE International Conference on Robotics and Automation (ICRA).

[5] Yuan C., Liu X., Hong X., Zhang F. (2021). Pixel-level extrinsic self calibration of high resolution lidar and camera in targetless environments. arXiv.

[6] Liu L., Stamos I. (2005). Automatic 3d to 2d registration for the photorealistic rendering of urban scenes. Proceedings of the 2005 IEEE Computer Society Conference on Computer Vision and Pattern Recognition (CVPR’05).

[7] Schneider N., Piewak F., Stiller C., Franke U. (2017). Regnet: Multimodal sensor registration using deep neural networks. Proceedings of the 2017 IEEE Intelligent Vehicles Symposium (IV).

[8] Zhu Y., Li C., Zhang Y. (2020). Online camera-lidar calibration with sensor semantic information. Proceedings of the 2020 IEEE International Conference on Robotics and Automation (ICRA).

[9] Zerveas G., Jayaraman S., Patel D., Bhamidipaty A., Eickhoff C. A transformer-based framework for multivariate time series representation learning. Proceedings of the 27th ACM SIGKDD Conference on Knowledge Discovery & Data Mining.

[10] Geiger A., Lenz P., Stiller C., Urtasun R. (2013). Vision meets robotics: The kitti dataset. Int. J. Robot. Res..

[11] Han K., Wang Y., Chen H., Chen X., Guo J., Liu Z., Tang Y., Xiao A., Xu C., Xu Y. (2022). A survey on vision transformer. IEEE Trans. Pattern Anal. Mach. Intell..

[12] Kramer O. (2013). K-nearest neighbors. Dimensionality Reduction with Unsupervised Nearest Neighbors.

[13] Zhao H., Jiang L., Jia J., Torr P.H.S., Koltun V. Point transformer. Proceedings of the IEEE/CVF International Conference on Computer Vision.

[14] Veličković P., Cucurull G., Casanova A., Romero A., Lio P., Bengio Y. (2017). Graph attention networks. arXiv.

[15] Rao Y., Zhao W., Zhou J., Lu J. (2022). Amixer: Adaptive weight mixing for self-attention free vision transformers. Proceedings of the 17th European Conference on Computer Vision.

[16] Hsiao T.Y., Chang Y.C., Chou H.H., Chiu C.T. (2019). Filter-based deep-compression with global average pooling for convolutional networks. J. Syst. Archit..

[17] Gao L., Chen L., Liu P., Jiang Y., Li Y., Ning J. (2024). Transformer-based visual object tracking via fine–coarse concatenated attention and cross concatenated MLP. Pattern Recognit..

[18] Liu H., Fang S., Zhang Z., Li D., Lin K., Wang J. (2021). MFDNet: Collaborative poses perception and matrix Fisher distribution for head pose estimation. IEEE Trans. Multimed..

[19] Kim J., Jang J., Park H., Jeong S. (2020). Structured consistency loss for semi-supervised semantic segmentation. arXiv.

[20] Ranasinghe K., Naseer M., Hayat M., Khan S., Khan F.S. Orthogonal projection loss. Proceedings of the IEEE/CVF International Conference on Computer Vision.

[21] Zhu J., Xue J., Zhang P. (2023). Calibdepth: Unifying depth map representation for iterative lidar-camera online calibration. Proceedings of the 2023 IEEE International Conference on Robotics and Automation (ICRA).

[22] Lv X., Wang B., Dou Z., Ye D., Wang S. LCCNet: LiDAR and camera self-calibration using cost volume network. Proceedings of the IEEE/CVF Conference on Computer Vision and Pattern Recognition.

[23] Iyer G., Ram R.K., Murthy J.K., Krishna K.M. (2018). CalibNet: Geometrically supervised extrinsic calibration using 3D spatial transformer networks. Proceedings of the 2018 IEEE/RSJ International Conference on Intelligent Robots and Systems (IROS).

[24] Fu Y., Li W., Fan S., Jiang Y., Bai H. (2023). CAL-Net: Conditional attention lightweight network for in-orbit landslide detection. IEEE Trans. Geosci. Remote Sens..

